# Cognition based bTBI mechanistic criteria; a tool for preventive and therapeutic innovations

**DOI:** 10.1038/s41598-018-28271-7

**Published:** 2018-07-06

**Authors:** Daniel Garcia-Gonzalez, Nicholas S. Race, Natalie L. Voets, Damian R. Jenkins, Stamatios N. Sotiropoulos, Glen Acosta, Marcela Cruz-Haces, Jonathan Tang, Riyi Shi, Antoine Jérusalem

**Affiliations:** 1 Department of Engineering Science, University of Oxford, Parks Road, Oxford, OX1 3PJ UK; 2 Weldon School of Biomedical Engineering, Purdue University, West Lafayette, IN USA; 3 Medical Scientist Training Program, Indiana University School of Medicine, Indianapolis, IN USA; 4 Oxford Centre for Functional MRI of the Brain, Nuffield Department of Clinical Neurosciences, University of Oxford, John Radcliffe Hospital, Oxford, OX3 9DU UK; 5 Army Registrar in Neurology and Lecturer in Medicine and Physiology, St Hugh’s College, St Margaret’s Rd, Oxford, OX2 6LE United Kingdom; 6 Centre for Functional MRI of the Brain, University of Oxford, Oxford, UK; 7 Present Address: Sir Peter Mansfield Imaging Centre, School of Medicine, and National Institute for Health Research (NIHR) Nottingham Biomedical Research Centre, Queens Medical Centre, University of Nottingham, Nottingham, UK; 8 Department of Basic Medical Sciences, College of Veterinary Medicine, Purdue University, West Lafayette, IN USA; 9 PULSe Interdisciplinary Life Science Program, Purdue University, West Lafayette, IN USA; 10 Purdue Institute for Integrative Neuroscience, Purdue University, West Lafayette, IN USA

## Abstract

Blast-induced traumatic brain injury has been associated with neurodegenerative and neuropsychiatric disorders. To date, although damage due to oxidative stress appears to be important, the specific mechanistic causes of such disorders remain elusive. Here, to determine the mechanical variables governing the tissue damage eventually cascading into cognitive deficits, we performed a study on the mechanics of rat brain under blast conditions. To this end, experiments were carried out to analyse and correlate post-injury oxidative stress distribution with cognitive deficits on a live rat exposed to blast. A computational model of the rat head was developed from imaging data and validated against *in vivo* brain displacement measurements. The blast event was reconstructed *in silico* to provide mechanistic thresholds that best correlate with cognitive damage at the regional neuronal tissue level, irrespectively of the shape or size of the brain tissue types. This approach was leveraged on a human head model where the prediction of cognitive deficits was shown to correlate with literature findings. The mechanistic insights from this work were finally used to propose a novel protective device design roadmap and potential avenues for therapeutic innovations against blast traumatic brain injury.

## Introduction

Blast-induced traumatic brain injury (bTBI), arising in particular from the exposure to improvised explosive devices, has become a major problem for the armed forces^1^ with mounting evidence pointing towards long-term neurodegenerative and neuropsychiatric disorders in veteran populations^2^. However, the link between the blast wave physics and the subsequent biological alterations in the brain remains largely elusive. Post-bTBI pathologies are thought to be mechanically initiated by the early-time propagation of stress waves^3^, cavitation effects^4^, rapid acceleration of the head resulting from shock wave and blast wind interaction with the body^5^, and secondary and tertiary injuries arising from subsequent impact, acceleration, and penetrating trauma^6,7^.

This work aims to provide new insights into the mechanical mechanisms of brain damage resulting from blast exposure by focussing on early-time events of primary blast loading and their correlation to post-injury biochemical and functional impairments. To this end, an *in vivo*/*in silico* methodology was developed to assess the consequences of blast on rats. We used a validated mild bTBI model in rats to expose individuals to blast conditions and, then, we performed a set of experimental techniques to characterise the distribution of oxidative stress reaction, a hallmark of secondary injury, across the brain and to relate these results to array of impairments in bTBI rats. The same blast conditions were then simulated in a validated *in silico* rat model allowing for determining the mechanical variables that govern brain damage resulting in cognitive deficits. After identification of the mechanical injury criteria that best correlate with oxidative stress and cognitive deficits, this approach was leveraged on an *in silico* human model addressing realistic blast conditions in the military context. The predictive brain damage regions were correlated to cognitive deficits finding a good agreement with impairments reported in the literature. The results have implications for strategies and approaches to diagnosis and treatment of bTBI, as detailed in the discussion. Furthermore, we provide new, validated bTBI *in silico* models of rat and human subjects capable of testing future innovations in combat head protection from blast and further exploring links between physical injury phenomena and downstream biological processes.

## Results

### Blast on rat: identification of mechanistic injury criteria in rat

#### Experimental programme

**Figure 1:**
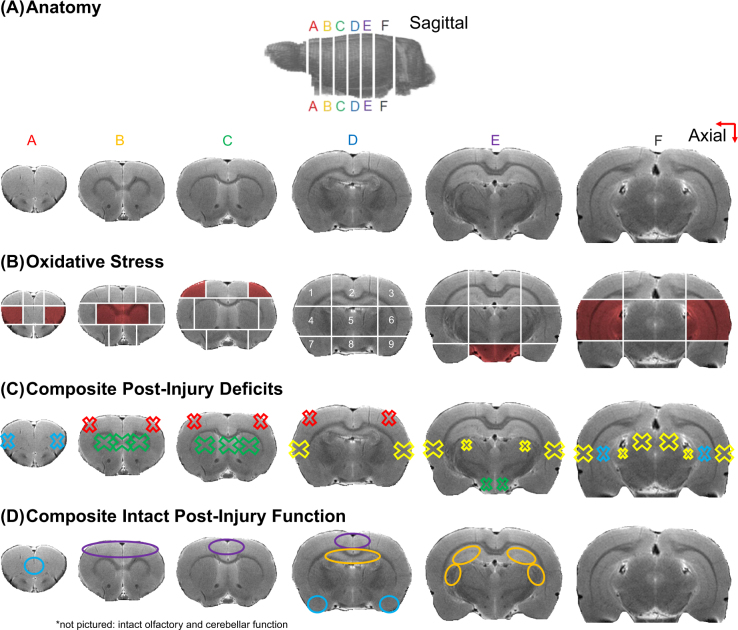
Summary of prior and current experimental findings in our rodent bTBI model: (**A**) T2-weighted MRI images illustrating the anatomy of the rat brain, axial section labelling A–F, and intra-section region labelling (1–9 in correspondence with Fig S1). (**B**) Spatial summary of significant oxidative stress elevations (red shading) observed *via* increased acrolein-lysine adducts on Western blot at 24 hours post-bTBI. (**C**) Composite presentation of observed post-bTBI deficits from 24 hours to 2 weeks post-injury including safety learning impairments related to orbitofrontal and hippocampal processing (blue X), Parkinsonian protein alterations in the striatum and midbrain (green X), facial and extremity pain (red X), auditory neurophysiological impairments in the auditory cortices, thalami, and inferior colliculi (yellow X). Each deficit was observed in separate cohorts of rats exposed to the same experimental blast exposure conditions used herein. (**D**) Spatial summary of regions thought to be intact after bTBI (equivalent performance to sham-injured rats) including medial prefrontal cortex and amygdala (blue O), motor cortices (purple O), rostral hippocampal areas (orange O), olfactory bulb and cerebellum (not pictured). Mild bTBI tissue damage and subsequent functional alterations can arise from i) direct mechanical injury to the brain in excess of one or multiple mechanical quantity thresholds and ii) inherent susceptibility of particular brain regions and/or cell types to post-injury secondary processes. The latter can manifest over an extended time scale (weeks-years) as post-injury degeneration unfolds. In the present investigation, we focus on the former and investigate the degree to which observed deficits in our rat bTBI experimental model could potentially be explained by mechanical perturbations incited by primary blast.

Previous experimental efforts summarised herein (Fig. 1A–C) were performed using a validated mild bTBI model in rats^8^. Consistent with clinical observations in human mild bTBI, our mild bTBI rats are acutely asymptomatic with unremarkable neuroimaging findings^8,9^. Despite the initial subclinical nature of our model, we observed empirical evidence that mild blasts can incite relative displacement of the rat brain with respect to the skull of the order of hundreds of microns to millimetres^10^. Consistent with other independent mild bTBI investigations^11,12^, we also report acute blood-brain barrier disruption^8^, downstream secondary oxidative stress elevations and neuroinflammatory alterations^8^ with later-onset behavioural and neurophysiologic alterations^9,13^.

First, we aimed to capture the spatial distribution of secondary injury processes across the brain during the peak secondary injury period, 24 hours post-injury^8^. To do so, we assessed the spatial distribution of acrolein adducts, a reactive aldehyde species with relatively long biological half-life frequently used by us and others as an indicator of sustained oxidative stress, one of the hallmarks of secondary injury^8^. We expanded our prior analyses^8^ and observed that, in a small minority of rat forebrain regions (8/53 regions), mild bTBI was associated with an increase in acrolein adducts (Fig. 1B). Other regions demonstrated minimal change with respect to sham-injured animals or highly variable measurements. When this distribution was compared to the array of impairments in bTBI rats, a reasonable degree of overlap can be observed (Fig. 1B,D). It is important to note that all prior studies and documented impairments were conducted under the same experimental blast exposure conditions used herein; thus, direct comparisons between current and past findings are appropriate. For example, increased oxidative stress, vis-à-vis acrolein adducts, was observed in the midbrain (Fig. 1B,C, region E8), where we have also observed post-bTBI Parkinsonian protein alterations^14^. Similarly, increased oxidative stress in the auditory thalamus and cortex (Fig. 1B,C, regions F4, F6) spatially correlates with our observed post-mild bTBI neurophysiological processing impairments in the same portions of the central auditory system^13^. Impairments in safety learning were noted as well^9^, corresponding to a combined functional alteration of the prefrontal cortex (Fig. 1B,C, regions A4&6) and hippocampus (Fig. 1B,C, regions F4&6), where we also observed an increase in oxidative stress in both regions. Additionally, brain regions related to cognitive deficits but not impacted by an increased oxidative stress (e.g., Fig. 1C, regions B1&3 and D1&3) were all indirectly connected to regions that were (Fig. 1C, regions C1&3). In contrast, regions with intact function/behaviour after bTBI generally did not overlap with areas affected by oxidative stress (Fig. 1B,D). This includes the bilateral motor cortex (Fig. 1B,D, regions B1–3, C2, D2) and amygdala (Fig. 1B,D, regions D7&9), which, consistently with the lack of deficits related to those areas^9^, did not demonstrate significant oxidative stress increases.

#### Numerical programme

**Figure 2:**
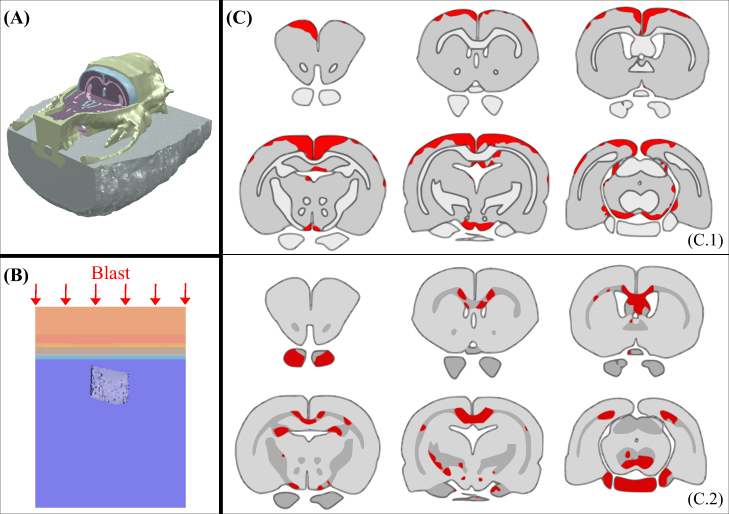
Numerical model of rat: (**A**) cut of a full FEHM presenting skin/fat, skull, CSF, grey and white matters; (**B**) blast loading imposed in numerical simulations; (**C**) brain injury predictions for shear energy rate criterion in grey matter (C.1) and for axonal stretch energy rate in white matter (C.2).

An important unresolved question is the mechanism by which an external head trauma induces the wide-spread patterns of oxidative stress reactions we observed in our rats. How the mechanical impact of bTBI on the brain is modulated by variations in local tissue properties cannot currently be evaluated *in vivo*. To this end, we aim at assessing correlative relationships between observed brain alterations and a range of mechanical variables by developing a detailed rat finite element head model (FEHM) from various MRI images (Fig. 2A). High-resolution anatomical images were segmented, and appropriate validated constitutive equations were associated to the segmented tissue types (*Supplementary Information*). In particular, novel constitutive models (*Supplementary Information* Fig. S5) were used for both grey and white matter to capture the differential responses of brain tissues under low- and high-rate loading (Equations (S.1–S.12)). The white matter model also incorporates tract orientation and fractional anisotropy acquired from diffusion-weighted images (Equations (S.7–S.9)). Consistent with *in vivo* experimental procedures, head fixations were applied in the *in silico* experiments, thus ensuring that the analysis of wave interactions from primary blast events is made in isolation from secondary and tertiary injuries (Fig. 2B). The model validation was performed against prior brain motion tracking experimental data^10^, see *Supplementary Information* Fig. S6. Using the validated model, numerous injury criteria were then assessed for spatial correlation with post-bTBI impairments (Figs 1 and 2) *a priori* linked to mechanical damage of both grey and white matters.

A set of literature-established and novel injury criteria was analysed for both grey and white matters: pressure, von Mises stress, equivalent strain, volumetric and shear energy rates. Note that the energy (as opposed to energy rate) sometime used in the literature as an injury criterion was not considered as a valid criterion as it can *a priori* reach high values in non-injuring, low frequency, low stress, long period loading. In the white matter, two complementary criteria were considered to account for mechanical anisotropy: axonal stretch and energy rate from axonal stretch (see *Supplementary Information* for further details). A summary showing the accuracy of each different criterion at predicting the damaged (Fig. 1C) and non-damaged (Fig. 1D) brain regions observed experimentally is presented in Table 1. The criterion best correlated with injured grey matter regions is the shear energy rate (damage threshold $\approx \,$100 MJ/m^3^s) at a matching accuracy of 72%. In the white matter, axonal deformation energy rate (damage threshold $\approx \,$1.5 MJ/m^3^s) was found to be the best criterion in terms of injury correlation, albeit with a relatively poor matching accuracy (56%). For all tested injury criteria, the predictive value was lower for white matter than grey matter. This finding supports indications that white matter deficits in the rat may be more likely to result from long-term secondary degenerative mechanisms mediated by glial and infiltrating peripheral inflammatory cells^15^. These predictions are supported by experimental observations in our own experimental mild bTBI model, after which these simulations were devised: i) unchanged post-TBI signal conduction speed between multiple relay stations ranging from the auditory nerve to the cortex^13^, suggesting intact myelination; and ii) intact resting-state functional connectivity between remote regions of a network involved in safety learning despite safety learning impairments^9^, suggesting that inter-regional projections were unharmed while intra-regional microstructural features suffered damage.

**Table 1:** Matching accuracy of the mechanical injury criteria based on the correlation of numerical results with experiments to predict damaged and non-damaged regions of brain.

Criterion	Matching accuracy (%)	
	Grey matter	White matter
Pressure stress	56	44
von Mises stress	39	33
Equivalent strain	56	44
Volumetric energy rate	56	22
Shear energy rate	72	44
Axonal stretch	—	33
Axonal stretch energy rate	—	56

#### Blast on human

**Figure 3:**
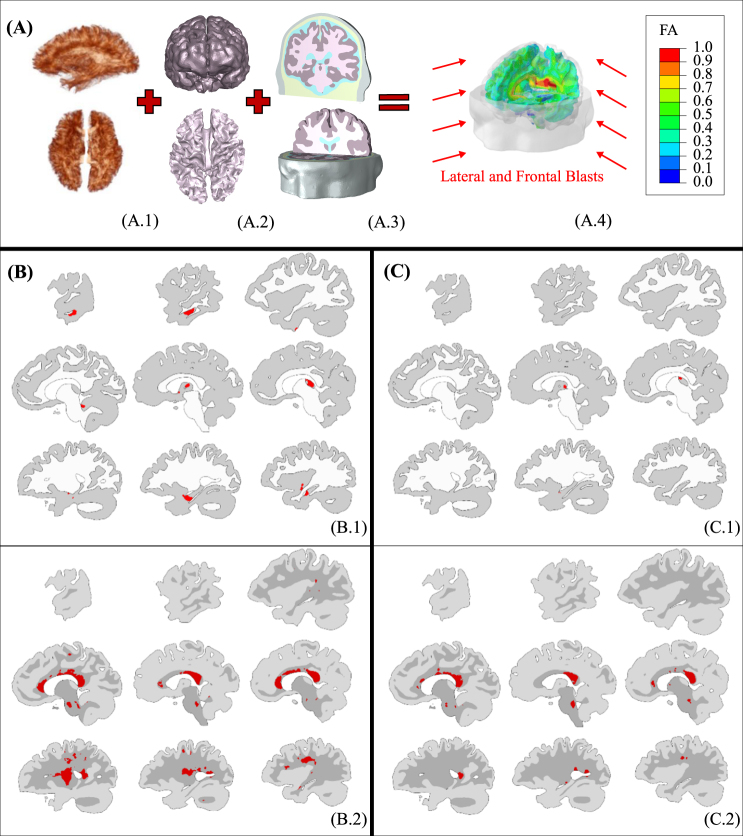
Numerical model of human head: (**A**) methodology based on the combination of axonal anisotropy from DTI (A.1), brain topology from MRI (A.2) and material properties for each head constituent (A.3) for the development of the full FEHM (here with fractional anisotropy heat map) (A.4); (**B,C**) brain injury predictions under lateral/frontal blast for shear energy rate criterion in grey matter (B.1/C.1) and for axonal deformation energy rate in white matter (B.2/C.2). The brain slices are presented from right to left.

The methodology developed for the rat FEHM was applied to the study of primary blast early-time wave interactions in the human head by identifying potential overlaps between areas injured in bTBI simulations and areas where damage is known to result in cognitive impairments in humans following bTBI^3^. The approach consists in leveraging the overall microstructural composition that gives rise to relative mechanical behaviours in white and grey matter^16^, and establishing inter-species mechanistic thresholds that correlate with cognitive damage at the tissue level irrespectively of the shape or size of tissue mesoscopic structures. For this purpose, the validated FEHM recently developed by Garcia-Gonzalez *et al*.^17^ was refined and human MRI Diffusion Tensor Imaging (DTI) information was incorporated to estimate the regional fibre orientation of white matter (Figs 3A and S4). The human head model was subjected *in silico* to four blast injury scenarios: frontal and lateral blast incident orientations at two blast intensities each: i) detonation of 2.3 kg charge of C-4 at a 2.3 m standoff distance (360 kPa magnitude blast) and ii) detonation of 3 kg charge of Octol explosive at 2–3 m standoff distance (1.3 MPa magnitude blast). Both scenarios are within the marginal limits for threshold lung damage but predicted to be survivable^18^. The lower intensity C-4 charge conditions did not breach the best correlated injury criteria thresholds obtained from the rat model (shear energy rate criterion for grey matter and axonal deformation energy rate for white matter), suggesting that brain injury from primary blast would not occur under these conditions. This is in agreement with previous observations^19^ suggesting that no serious cerebral contusions are expected under 379 kPa. Under the more intense Octol charge conditions, however, numerical results predicted primary blast-induced injury in numerous brain regions (Fig. 3B,C).

The lateral-incident blast simulations predict acute grey matter damage in the ipsilateral cerebellar cortex (lobule III/IV) and inferior/middle temporal gyrus, bilateral thalami, and contralateral hippocampus, see Fig. 3B.1. Predicted white matter disruptions (Fig. 3B.2) are localised to the corpus callosum and corticospinal tract, with additional involvement of the cerebellar peduncles, anterior thalamic radiations, lateral and dorsal segments of the superior longitudinal fasciculus pathways and sagittal stratum. Despite the much lower degree of confidence of the proposed white mater injury criterion in the rat, the overall pattern of predicted tissue injury correlates well with many regions known to be vulnerable to blast, namely the thalamus^20^, hippocampus and cerebellum^21^, as well as the white matter fibres of the cerebellar peduncles and internal capsule^22^. Disruption of additional fibre pathways varies across blast trauma survivors^23,24^ and, according to our results, may at least partially reflect differential effects of blast injury depending on head orientation and blast intensity. Indeed, the frontal-incident blast wave simulations (Fig. 3C) induce focal injuries in a different distribution than is observed for the lateral-incident blast. While a frontal blast is also predicted to injure the thalamus and corpus callosum, other brain areas differ in terms of injury severity. The observed multi-focal injury distribution patterns with dependence on blast incident orientation is consistent with prior reports^3^. Previous investigations implemented stress, strain, or pressure-based injury criteria and predicted primarily coup-contrecoup injury patterns^3,25^. However, our predictions based on shear energy rate (grey matter) and axonal energy deformation rate (white matter) suggest a largely periventricular injury distribution focussed around the centre of the brain. Note that our results showed shear concentration in different brain regions under blast exposure. Contrary conclusions have been suggested for this type of loading^7^. This difference comes from the consideration of different components within the brain (grey/white matter and ventricles) that results in shear accumulation on the material boundaries. These observations are in agreement with the work of Taylor and Ford^3^, where focal shear concentrations in grey-white matter junctions for early-time wave interactions were found.

## Discussion

The pattern of injury in bTBI is likely to determine the ensuing cognitive and psychiatric problems suffered by injured humans and seen in experimental models. The ability to predict such patterns is, therefore, a crucially important component of bTBI models, especially those designed to test the likely benefit of head protection. Our human FEHM predicted that periventricular brain regions, particularly the thalamus and corpus callosum, may be vulnerable to early-time events during blast exposure in an orientation-independent manner. Abnormalities in the thalamus are a consistent feature of TBI, likely due to its dense cortical interconnections. PET studies suggest that thalamic abnormalities represent long-term markers of TBI, in particular thalamic neuroinflammation that, interestingly, co-localises with axonal damage^26^. Severity of thalamic injury has been proposed as a potential predictor of various clinical and neurocognitive outcomes following TBI^20^. In addition, the corpus callosum has long been hypothesised as a vulnerable substrate for mechanical disruption in TBI and has been observed to be compromised in both rodent^27^ and human^28^ bTBI. Notably, DTI reveals significant, localised fractional anisotropy reductions in the corpus callosum in veterans with a history of blast-exposure compared to those without^28^, while other brain regions remain ostensibly unaffected. Additionally, it is well recognised in TBI among other conditions that the precise focus of injury often only partially explains patient symptoms. Instead, damage to the brain’s major white matter connection pathways has been consistently linked to post-injury performance declines on cognitive measures including executive function and associative memory^29^. These deleterious effects of white matter disruption are thought to arise from disruption to widely distributed functional networks^30^. Both the thalamus and corpus callosum are positioned centrally with widespread, cross-brain connectivity which, if damaged, could have profound implications on normal brain function. Resultant dysfunction from white matter damage to these structures could manifest in numerous ways, ranging from discrete neurological deficits to network-scale dysfunction. This could appear clinically with changes as simple as altered sensory processing secondary to discrete lesions of white matter tracts (e.g., a lesion exclusively affecting thalamic relay afferents to the auditory cortex). The contribution of distinct patterns of cortical and white matter damage to emotional processing in TBI has received less attention. However, recent data indicate that damage to white matter connections, especially in frontal lobe pathways, differentiates TBI patients with and without mood disorders^31^. The impact of tract-specific injury may, however, be more nuanced, as further evidence indicates that both white matter and functional MRI measures of cortical synchronisation independently predict post-traumatic stress disorder severity^32^. Together, these emerging findings lend support to the possibility that blast-induced damage to inter-regional axonal projections would impair coordination of synchronised activity across brain regions within a given network, resulting in functional impairments.

Model-predicted injuries to brain regions outside the thalamus and corpus callosum vary with exposure conditions, consistent with the wide range of post-bTBI clinical presentations and known variability in fibre tract involvement among bTBI survivors^23,24^. These injuries may also manifest as isolated neurological deficits or disruption of network-scale coordinated activity, per our discussion above. Persistent cognitive deficits following head trauma most often involve memory, attentional set-shifting and processing speed^33^. Predicted damage to the vulnerable fibre tracts was shown to significantly predict variance in executive functions and processing speed in TBI patients^34^. Additionally, disruption of hippocampal networks offers a recognised mechanism for deficits in learning and memory. Recently, dysfunction of the anterior hippocampus, implicated in emotional memories^35^, has been linked directly with severity of post-traumatic stress disorder in veterans^36^. Focal damage to the anterior hippocampus observed in our lateral blast model, and in previous human postmortem samples^2^, therefore, offers a candidate substrate for both memory and chronic emotional disorders prevalent after TBI^22^. Predicted damage to both the anterior cerebellum, containing projections of the sensorimotor system^37^, and corticospinal fibres in the immediate aftermath of blast wave exposure likely account for occasional, usually transient^21^, motor deficits noted in murine and non-human primate^38^ blast trauma models.

An important question arising from this work is whether brain injuries resulting from blast events differ significantly from ‘conventional’ non-blast TBI incited by impact-acceleration events. This work points towards the conclusion that blast and impact-acceleration injuries do mechanistically differ at multiple scales because of three key factors: injury location, time scale of injury, and co-occurrence. The most common blast injury locations were at material boundaries (brain-CSF or grey-white matter, consistent with independent predictions^39,40^) near the centre of the brain, whereas conventional TBI classically presents in coup-contrecoup, diffuse axonal injury, and rigid-boundary-adjacent distributions^41^. Conventional TBI injury distributions have historically been quite well-predicted by traditional stress and strain injury criteria^42^, which performed poorly in predicting injury in our bTBI model in comparison to shear and axonal stretch energy rates. Prior to this work, it was unknown whether experimental blast-induced mechanical injury profiles bear any relationship with preclinical ‘outcomes’ – ranging from biochemistry to behaviour – which we now suggest is the case. Broadly, this means that even in the absence of longer time-scale impact-acceleration injuries, high-rate early time (order of microseconds) mechanical injuries from blast loading can cascade into relevant physical damage to brain tissue which correlates with experimental endpoints. It is well established that CNS injuries are loading rate- and magnitude-dependent^43,44^. It is a logical extension that blast-type loading, which occurs over a much shorter time scale compared to conventional (impact) TBI (microseconds *vs.* milliseconds) and induces loading at higher strain rates, should thus differ in the resultant injury profile to cells and tissue. We have recently predicted some mechanical effects of such high-rate blast injuries at the cellular^45^ and molecular level^46^, but supporting experimental data is lacking in the current body of bTBI literature. It is important to note that blast and conventional TBI often (though not exclusively) occur together, and as such may be difficult to distinguish clinically in many cases due to limitations of current diagnostic tools. Despite this, mindfulness of differential injury mechanisms between blast and conventional TBI, particularly with respect to injury locations and loading rates, can guide future efforts toward mechanobiological understanding as well as diagnostic and therapeutic innovations.

Relationships between microstructural physical damage, corresponding mechanical thresholds for cognitive, emotional and neuromotor deficits, and subsequent recovery or degeneration within injured areas offer important avenues for further research. The proposed approach allows for the prediction of cognitive deficits in rats submitted to early-time blast effects with an indication that white matter damage mainly arises from secondary effects in the rat. Our human predictions are aligned with literature findings of both white and grey matter damage. The difference between rats and humans in white matter early-time injury might be linked to a different colocation of reached mechanical thresholds and molecules associated with neuroinflammatory processes, and/or might be explained by the structural differences between lissencephalic and gyrencephalic brains^47^. TBI pathophysiology is known to exist on a continuum initiated by primary (physical) injury consisting of disruption to cellular structures, that then progresses *via* secondary (biochemical) mechanisms which culminate in neuronal dysfunction, degeneration, and ultimately clinical deficits^48^. Widely reported secondary injury mechanisms include oxidative stress, excitotoxicity, and neuroinflammation^48^. Interceding mechanisms linking primary and secondary injury mechanisms remain, however, poorly understood. Mechanosensitive proteins are putative mediators of the transition from primary to secondary injury processes. Transient receptor potential (TRP), two-pore domain potassium (TREK, TRAAK), and aquaporin (AQP) channel families all represent mechanosensitive protein targets for future research. Each group contains member proteins which are highly expressed in the brain, specialised for sensation of and response to mechanical perturbations, and have been linked to downstream neuroinflammatory processes^49–51^. Future work targeting the effects of modulating these proteins on post-TBI secondary injury processes and outcomes should be explored to fully dissociate early-time physical damage from secondary injuries.

Beyond future research into pathophysiological mechanisms of bTBI, the findings herein have implications for both preventative and therapeutic innovations.

### Preventative innovation in protective devices

In the military context, prevention is multifactorial, influenced by personnel training, pre-operative military intelligence, combat tactics and protective technology. To date, improvements in helmet design have reduced the number of penetrating injuries, but their effect on outcomes following bTBI have been less pronounced. In part, this results from decreases in wearability when adjustments are made to helmets with the aim of protecting against blast. For example, increased padding adds additional weight, whilst visors capable of dissipating blast wave energy can restrict both head movement and visibility. Further helmet design, using novel materials and shapes, is urgently needed. However, a suitable model of bTBI is crucial to the design process. To that end, the work presented here has the potential to provide significant advances in the future development of bTBI protective equipment. By identifying the mechanical variables governing damage in the rat brain, transferring them to the human brain model, and predicting injury in those brain regions known to be damaged by bTBI, our approach enables the guided design of new protective devices against blast. It does so through: i) the study of the relationship between the protective device mechanical properties and the resulting bTBI protection; ii) a complete study of the protective capability against bTBI of final prototypes according to the new cognition-based mechanistic criteria presented. We present here an analysis of i) and provide the basis, tools and methodology for further investigation in terms of ii).

**Figure 4:**
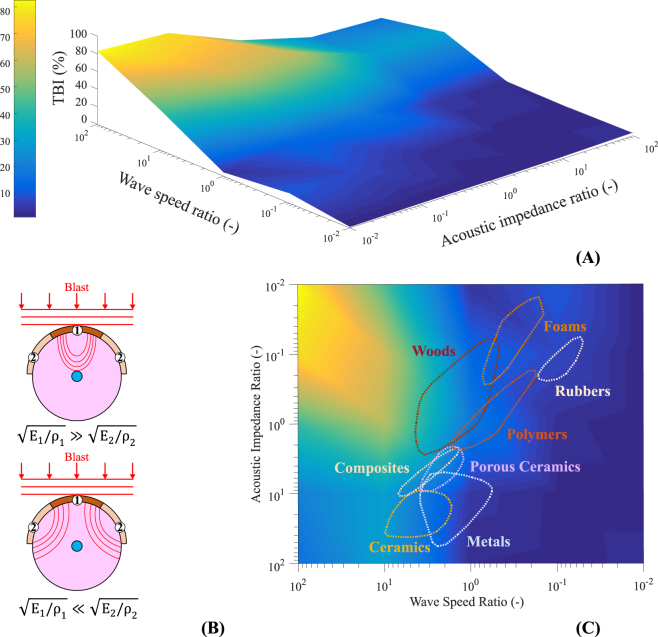
(**A**) Proportion of brain region reaching the shear energy rate threshold of 100 MJ/m^3^s (TBI) for different acoustic impedance ratios between shield components ($\sqrt{{{\rm{E}}}_{2}{\rho }_{2}}/\sqrt{{{\rm{E}}}_{1}{\rho }_{1}}$) and wave speed ratio between shield components ($\sqrt{{{\rm{E}}}_{2}/{\rho }_{2}}/\sqrt{{{\rm{E}}}_{1}/{\rho }_{1}}$). The mechanical properties of polycarbonate (used in visors of blast protective helmets) are assumed for ${{\rm{E}}}_{1}$ and ${{\rm{\rho }}}_{1}$. (**B**) Schematic pressure wave patterns within brain tissue depending on the shield material composition (assuming constant impulse mitigation and acoustic impedance). (**C**) Wave speed ratio *vs.* acoustic impedance ratio map for polycarbonate visor helmet materials ➁ selection (➀ is polycarbonate).

To analyse the dependences of bTBI protective capability on the material properties of protective devices, a simplified FEHM was designed as a combination of concentric spheres representing: skull, CSF, brain and ventricles. The homogenised brain tissue was assumed isotropic for this purpose using the grey matter constitutive model developed herein, which incorporates the shear energy rate criterion. An additional layer was incorporated representing a protective shield on the head. The protective role played by the shield relies on its ability to influence three aspects of the transmitted stress wave: a reduction in the stress amplitude; impulse mitigation; and a change in the stress wave shape^52^. Therefore, the optimal design of protective devices must take into account these three characteristics of the stress wave transmitted to the head and, subsequently, to the brain tissue. The reduction in the stress amplitude can be controlled through the acoustic impedance of the shield material $\sqrt{{{\rm{E}}}_{{\rm{s}}}{{\rm{\rho }}}_{{\rm{s}}}}$, where ${{\rm{E}}}_{{\rm{s}}}$ is the Young’s modulus of the shield and ${{\rm{\rho }}}_{{\rm{s}}}$ is the density of the shield (see *Supplementary Information* for further details). Impulse mitigation can be reached by increasing the shield mass and/or by additional consideration of inelastic deformation in the shield (affecting ${{\rm{E}}}_{{\rm{s}}}$). Here, we focus particularly on the design of shields involving different structural materials resulting in regional variations of the wave speed and acoustic impedances. The variations in wave speed induce changes in the shape of the stress wave propagated within the brain, which we aim at leveraging for increased protective efficacy of the shield. To this end, with the aim of decreasing the large accumulation of stress, and eventually the increase of shear energy rate, around the ventricles in the centre of the head, the shape of the stress wave transmitted to the brain can be modulated by introducing a wave speed gradient along the shield by controlling $\sqrt{{{\rm{E}}}_{{\rm{s}}}/{{\rm{\rho }}}_{{\rm{s}}}}$ (see Fig. 4B). Note that a variation in wave speed implies the variation of the acoustic impedance, the transmitted impulse or both.

In this study, the acoustic impedance and the variation of the stress wave shape were analysed. As a preliminary design for the shield, two regions with different material properties are considered. The central region (① in Fig. 4B, with a ratio of 1/3 between the central region diameter and the total shield diameter) is chosen to be polycarbonate, a material commonly used in the visor of blast protective helmets^53^. The Young’s modulus and density of region ② were varied accordingly to analyse their influence on the transmitted stress wave. The different cases were simulated using the finite element solver Abaqus/Explicit. A VUMAT subroutine was used to define the brain behaviour as homogenised and isotropic, incorporating the shear energy rate damage variable. The threshold obtained from the rat simulations (100 MJ/m^3^s) was used to compare the different cases in terms of damage patterns. The blast loading imposed was that used in the human simulations resulting in bTBI. The simulations show that the extent of damage in the brain region strongly depends on the shield’s mechanical properties. In this regard, a reduction in the wave speed (lower wave speed ratios in Fig. 4A) or an increase of acoustic impedance in the shield (higher acoustic impedance ratios in Fig. 4A) both result in an improvement in terms of blast protection. The influence of the acoustic impedance ratio and the wave speed ratio between shield regions, and the interplay between both on the effectiveness of the protective device is presented in Figs 4A and C. In Fig. 4C, the regions covered by polycarbonate-based shields for different materials accompanying the polymer are overlapped. The results for the specific geometry employed show optimal solutions for shields that combine polycarbonate visor with metals or foams shells. The most obvious missing considerations are i) the weight and ii) the possibility to multilayer the shield, both leading to the consideration of additional dimensions in the optimisation process.

Overall, the numerical tools and the methodology developed herein provide the basis for further optimisation of protective devices to avoid bTBI. Efforts toward innovative protective technology should be experimentally tested and validated. Such advancements, while beneficial and necessary, are unlikely to completely obviate the need for additional TBI intervention.

### Therapeutic innovation in TBI treatment

As discussed above, the post-injury physiological course of TBI pathology is inherently complex, dynamic, and features numerous biological mechanisms whose prominence varies with post-injury latency^54^. Symptomatic treatments with pharmacologic or rehabilitative measures have shown promise^55–57^, but efforts directed toward pharmacologic neuroprotection have yet to reliably demonstrate clinical efficacy despite the robust preclinical success of numerous compounds^54^. Of interest for the present results, we and others have documented correlative relationships between oxidative stress and post-TBI outcomes^9,58–60^; anti-oxidant therapies have demonstrated early promise pre-clinically (for review, see^61^); and some anti-oxidant molecules have recently progressed to clinical trials^62,63^. More recently, reactive aldehydes (acrolein, 4-HNE, MDA, and related compounds) have emerged as novel pharmacological targets to reduce oxidative stress. Such molecules are of particular interest due to our prior work studying aldehyde-mediated effects in spinal cord injury and multiple sclerosis^64–66^, in addition to independent links between reactive aldehydes and neurodegenerative diseases^67^. Aldehyde reduction therapies are still in early stages of development and testing, but show promise for brain injuries of both ischemic and traumatic aetiology^68–70^.

Ultimately, pairing anti-oxidant/anti-aldehyde therapies with interventions targeted at complementary mechanisms of injury (mechanical disruption, excitotoxicity, inflammation, etc.) are likely to provide the greatest chances for success^54,57^. However, choosing which interventions to give and when to give them remains an ongoing clinical challenge. As our results suggest, it is possible and likely that both exposure-independent injuries (thalami and corpus callosum) and exposure-dependent injuries (cortical areas) occur following blast-induced TBI. This could have significant implications for treatment targeting. If our data are validated with future investigations, the results would suggest interventions found to be efficacious for thalamic and callosal injuries could potentially be provided empirically, while interventions to prevent/treat cortical deficits may benefit from patient selection procedures and more in-depth diagnostic workup. Given the large-scale clinical trial failures of efforts at global neuroprotection in TBI patients^54^, it is conceivable that injury to different brain regions may require disparate therapies suited to affected areas that are individualised to each patient. At present, clinical care in most settings does not stratify TBI treatment by injury location and would treat such injuries similarly according to current standards of care. This is in part due to the fact that standard clinical imaging assessments (CT and basic MRI) detect only areas of significant oedema or haemorrhage^71^ and cannot identify stretched axons, micro-scale disruptions of brain architecture, inflammation, or subtle changes in brain function. This is especially problematic in mild TBI, when injuries typically cannot be visualised with standard CT or MRI modalities^71,72^. As such, refinement of clinical assessment tools to evaluate the location/extent of injured intracranial tissue and accompanying functional loss is essential for the advancement of personalised, brain-region-specific medicine in the care of TBI.

Techniques for early identification of cortical injury location and severity to support patient selection and targeted delivery of therapy could include: FEHM simulation-aided injury reconstruction, clinical neuropsychomotor assessment, neurophysiologic testing, advanced neuroimaging, and serum or CSF biomarkers. These are all areas of active research, but further validation is needed before clinical utility is reached. As diagnostic technologies improve in accuracy and accessibility, the study and treatment of brain injuries should account for location of injury in the design of treatment strategies. Herein we have focussed on mechanical contributors to injury location, but it is also important to consider innate biological diversity in regional/cellular ultrastructure, inherent metabolic distinctions between cell types, and discrepant expressions of cell-survival-related genes and proteins. Variations in these parameters lend distinct brain regions and cell types therein to exhibit differential susceptibility under similar injurious conditions^73^.

Brain injuries that supersede our ability to prevent will benefit from personalised treatment strategies, the considerations and complexities requisite for which are vast. Using a novel approach attempting to correlate early phase blast-induced physical phenomena with later damages and deficits on the order of hours to weeks, we have generated results which support consideration of injury location in treatment strategy design. If confirmed, these results influence therapy application and development and, further, necessitate rapid advancement in diagnostic tools for patient stratification to guide clinical decision-making.

## Methods

### Experimental methods

This section summarises the new experimental techniques used in this work while details on the experimental methods used for brain injury exposure and brain deformation recordings (previously published by the authors) can be found in *Supplementary Information*.

### Biochemical analysis of acrolein-lysine adducts to assess brain oxidative stress

Classic Western blotting (WB) techniques were utilised to assess acrolein-lysine protein adducts, a reliable marker of oxidative stress in neural trauma and degenerative disease^8^, in the rat brain following the mild blast exposure described above^9,14^. Rat brains were perfused with cold, oxygenated Krebs solution and removed at 24 hours following injury, the time at which acrolein levels peak in the brain after exposure to this mild bTBI^8^. After transcardial perfusion, the whole brains were quickly removed and flash frozen with dry ice and stored at −80° C until processing. Microdissected regional lysates were obtained in six consecutive coronal sections, starting just caudal to the olfactory bulb. The coronal sections were each subdivided with two frontal cuts and two sagittal cuts (forming a 3 × 3 grid) into nine distinct regions per section. Coronal sections were cut at the following locations with respect to Bregma: + 4, + 2, 0, −2, −4, −6, and −9 mm into sequential sections labelled A–F (i.e., section A ranges from +4 to +2 mm)^74^. Viewed from the anterior of each section, within-section segments were numbered viewer left (subject right) to viewer right (subject left), top (dorsal) to bottom (ventral) such that segments 1, 4, and 7 generally correspond to right hemisphere neocortex; 3, 6, and 9 generally correspond to left hemisphere neocortex; 2 corresponds to midline dorsal neocortex; 5 and 8 correspond primarily to subcortical regions (i.e., region A5 corresponds to the centre region of the rostral-most section). Dissected regions were homogenised and sonicated in 1 × RIPA buffer (Sigma-Aldrich, catalogue #: R0278) with added protease inhibitor cocktail (Sigma-Aldrich, catalogue #: P8340) diluted to a 1:100 final concentration. Centrifugation of samples was performed at 15,000 g for 40 minutes at 4 °C. Supernatant was extracted for protein quantification and electrophoresis.

Prior to Western blotting, protein concentrations were measured using the Bicinochoninic Acid (BCA) protein assay kit (Pierce) and quantified on a SPECTRAmax raw optical density plate reader (Molecular Devices). 60 µg protein with 20% SDS, β-mercaptoethanol, and 2 × Laemmli buffer were loaded into 15% Tris-HCL gels and electrophoresed at 80 volts for 2–3 hours. Protein lysate was then transferred to nitrocellulose membranes via electroblotting in 70 volts for 1 hour in 1 × transfer buffer with 20% methanol (BioRad, Tris-Glycine buffer). Blocking with 1 × casein (Vector, catalogue #: SP-5020) was performed for 1 hour at room temperature. Primary anti-acrolein antibody was used (Abcam, catalogue #: 37110) for overnight incubation. Incubation with anti-mouse antibody (Vector, catalogue #: BA-2000) was then conducted for 1 hour at room temperature. DuoLux substrate immundetection kit (Vector, catalogue #: SK-6605) was used for chemiluminescent signal acquisition of the blots on an Azure c300 imaging system (Azure Biosystems). Band intensity (entire lane for acrolein-lysine adducts) was quantified in AlphaView software using local background averaging. Protein of interest bands were normalised with β-actin from the same sample. Analysis results (see *Supplementary Information* Fig. S1) are present as ratios with respect to sham-injury levels in the same anatomical areas. Sub-analysis of six regions (A2, A4, A5, A6, B7, and B9) was presented previously^9^; these results are re-illustrated here for completeness.

### Behavioural analysis of motor, sensory, cognitive, affective, and psychosocial function

Motor, cognitive, affective, and psychosocial behavioural assessments after mild bTBI in this model after animals were subjected to the same blast exposure conditions used herein have been documented previously as summarised in Fig. 1^8–10^. Myofacial hypersensitivity was newly assessed as part of the present investigation (see *Supplementary Information* Fig. S2). Behavioural testing for mechanical periorbital allodynia was assessed by facial withdrawal thresholds in response to a series of von Frey filaments with calibrated bending forces according to the size of the filament (range: 0.01–11 g, Stoelting, Wood Dale, IL, USA). Allodynia refers to the abnormal painful responses to stimuli that are normally not painful; and allodynia testing with von Frey filaments in rodents is a well-known and validated method to evaluate sensory changes after CNS injury^75,76^. The rats were tested using a universal plastic tube restraint designed for rodents up to 500 g (Stoelting, Wood Dale, IL, USA); and restrained without force for less than 15 minutes, including 5–10 of acclimation and 5 minutes of testing. A behavioural response was indicated by a sharp withdrawal of the head. The rats were tested for mechanical periorbital allodynia twice before induction of mbTBI to establish a baseline withdrawal threshold, and again 8 days post-blast.

### Numerical methods

#### Finite element head models: mesh generation from MRI and axonal incorporation from DTI

In this work, detailed rat and human finite element head models (FEHMs) were developed from high resolution MRI images of a subject. These images were first segmented into skin/fat, skull, cerebrospinal fluid (CSF), grey matter and white matter and then meshed using Amira software. The pre-processing of the head model without the axonal tract information follows the previous work of Garcia-Gonzalez *et al*.^17^. Briefly, geometrical information was obtained from high resolution anatomical T1 and T2-weighted MRI images of Subject ID: 100307 of the Human Connectome Project^77^. Inner skull, outer skull and outer scalp surfaces were extracted by use of “BET2”^78,79^ in the FSL software library^78^ and the organ segmentation was performed by using the Amira software. The defaced MRI images by the HCP were manually reconstructed in our model without affecting the brain^80^. The resulting mesh for the rat, after verification of spatial convergence, is made of 3,408,851 tetrahedral elements and weights 21.9 g (see *Supplementary Information* Fig. S3). For the human, it is made of 2,354,594 tetrahedral elements and weights 3.91 kg (see *Supplementary Information* Fig. S4).

A novel approach was applied for the definition of CSF. Since CSF is mechanically considered as incompressible, a standard finite element Lagrangian definition of the CSF mesh was observed to result in a locking of the individual CSF finite elements, effectively blocking the relative displacement of the brain with respect the skull. In order to allow for the flow of CSF fluid and the subsequent relative displacement between brain and skull (observed in our experiments), this part was defined instead by using smoothed particle hydrodynamics (SPH) elements. SPH is a meshless Lagrangian method that guarantees the conservation of mass and computes the stress and deformation gradient from weighted contributions of neighbouring particles while allowing for the incompressibility condition of the CSF as a whole and without restricting the fluid flow^81^.

HCP diffusion MRI (dMRI) preprocessed data was used to estimate the axonal orientations within the white matter^82^. Node locations for the brain white matter surface mesh were exported and superimposed in space on the acquired fibre orientation and fractional anisotropy (FA) data (custom Matlab script). Voxelwise extraction of 3-axis orientation and FA scalar value information was performed for each node (custom Matlab script). The preferred axon orientation ${\hat{{\bf{a}}}}_{{\bf{o}}}$ and a FA coefficient that represents the degree of anisotropy could then be computed for each element^83^. Details on the acquisition of anatomical and diffusion-weighted magnetic resonance images of the rat brain as well as on the alignment and processing of diffusion-weighted images are provided in *Supplementary Information*.

This anisotropic information of the brain mechanics was linked with the FEHMs through the dMRI dependent constitutive modelling of white matter in a VUMAT material subroutine for the finite element solver Abaqus/Explicit^81^. Both Lagrangian/SPH FEHMs were embedded into an Eulerian mesh for the surrounding air through which the shock wave propagates.

#### Mechanical behaviour of biological tissues

The prediction of stress wave propagation in solid materials requires the definition of both bulk and shear responses. In this work, a formulation based on the decomposition of the stress response into volumetric and deviatoric (isochoric) components is proposed as:

1 $${\boldsymbol{\sigma }}=-\,{\rm{P}}{\bf{I}}+{{\boldsymbol{\sigma }}}_{{\bf{i}}{\bf{s}}{\bf{o}}}$$

where ${\boldsymbol{\sigma }}$ is the Cauchy stress tensor, P is the pressure (positive in compression), **I** is the second order identity tensor and ${{\boldsymbol{\sigma }}}_{{\bf{i}}{\bf{s}}{\bf{o}}}$ is the deviatoric component of the Cauchy stress tensor.

The deformation of the material is characterised by the deformation gradient tensor which is multiplicatively decomposed into volumetric and deviatoric components:

2 $${\bf{F}}={{\rm{J}}}^{1/3}{{\bf{F}}}^{\ast }$$

where ${\rm{J}}=\,{\rm{\det }}({\bf{F}})$ is the Jacobian and ${{\bf{F}}}^{\ast }$ is the distortional part of the deformation gradient.

Taking as basis the constitutive framework provided by Garcia-Gonzalez *et al*.^84^ together with the previous expressions for the stress and deformation gradient, the constitutive laws ${\boldsymbol{\sigma }}({\bf{F}})$ that describes the mechanical behaviour of grey and white matter of brain, as well as details on the constitutive modelling that describes the mechanical behaviour of the remaining biological tissues and the surrounding air can be found in *Supplementary Information*. These constitutive models have been implemented in a VUMAT material subroutine for Abaqus/Explicit and assigned to the corresponding parts of the model (with exception of the constitutive model for CSF and ventricles that is already implemented in Abaqus).

#### Injury criteria

Many authors have suggested the use of stress and strain based variables such as pressure, von Mises stress or equivalent strain for the definition of brain injury thresholds^7,17,85^. In the white matter in particular, axonal stretch is commonly used as the main strain based criterion^86,87^. Other studies have proposed energy based criteria to associate damage with high stress levels only if a significant deformation is induced^88^. Since strain rate was demonstrated to play a significant role in brain injury and noting that energy is in fact a time cumulative quantity (and thus depends on the overall time of an event), we propose here instead an additional criterion based on the maximum rate of energy (or power) reached during the deformation process. In this work, we simultaneously considered the identified injury in order to determine which one(s) best correlates brain primary injury (as identified through oxidative stress) under blast conditions. For grey matter, we considered pressure, von Mises stress, equivalent strain, and volumetric and shear energy rates. For white matter, two complementary criteria have been also considered accounting for the axon-induced anisotropy: axonal stretch and energy rates of the axonal stretch deformation. For more details on the injury criteria definition see *Supplementary Information*.

#### Validation of the numerical model

The experiments conducted by the authors in a previous work provided the displacement of a soft magnet implanted on the surface of the brain in living rat, dead rat and 3D printed rat brains during blast exposure^10^. Consistent with *in vivo* experimental procedures, head fixations were applied in the *in silico* experiments, thus ensuring that the analysis of wave interactions from primary blast events is made in isolation from secondary and tertiary injuries. A validation of the full rat FEHM model was then performed by comparing its predictions of the brain displacement during the blast against the experimental tracking. *Supplementary Information* Fig. S6 shows the comparison of the model predictions for the total displacement with experiments from the time when the inertial effects start to play an important role. A good agreement was found with the model predictions. In addition, the numerical brain displacement was predicted to be between the experimental results obtained for the live rat, the dead/3D printed rat. Note that as the implanted soft magnet moves away from the reference transducer on the skull during the blast, the system has an innate tendency to overestimate displacement, as described previously^10^, and the degree of overestimation increases the further away the magnet moves. The results, along with this observation, suggest that the numerical model is able to capture the physics of the blast exposure. The validated model can be then used to identify the mechanical variables that govern the injury process in brain.

### Guidelines and Regulations Statement

All animal experiments were reviewed, approved, and conducted under animal use protocols overseen by the Purdue Animal Care and Use Committee (Protocol #1111000280).

### FEHM meshes

The FEHM meshes (*Finite Element Human Head Model* and *Finite Element Rat Head Model*) can be downloaded on http://jerugroup.eng.ox.ac.uk/fehm.html and the Oxford University Innovation Software Store https://process.innovation.ox.ac.uk/software.

### Patent

Subject matter from this paper forms part of Patent Application No. GB1716849.3 entitled “Protective Device” filed on 13 October 2017 in the name of Oxford University Innovation Ltd.

## Electronic supplementary material

Supplementary Information
