# Supplementary material for: Cognition based bTBI mechanistic criteria; a tool for preventive and therapeutic innovations
Source: Sci Rep. 2018 Jul 6;8:10273. doi: 10.1038/s41598-018-28271-7 (PMC6035210; doi:10.1038/s41598-018-28271-7)
Supplement: Supplementary file 1 — Supplementary Information [file 41598_2018_28271_MOESM1_ESM.pdf]

Supplementary Information

Cognition based bTBI mechanistic criteria; a tool for preventive and therapeutic innovations

†Daniel Garcia-Gonzalez[1], †Nicholas S. Race[2,3], Natalie L. Voets[4], Damian R. Jenkins[5], Stamatios N Sotiropoulos[6,7], Glen Acosta[8], Marcela Cruz-Haces[2], Jonathan Tang[2], *Riyi Shi[2,8,9,10], *Antoine Jérusalem[1]

[1] Department of Engineering Science, University of Oxford, Parks Road, Oxford OX1 3PJ, UK

[2] Weldon School of Biomedical Engineering, Purdue University, West Lafayette, IN, USA

[3] Medical Scientist Training Program, Indiana University School of Medicine, Indianapolis, IN, USA

[4] Oxford Centre for Functional MRI of the Brain, Nuffield Department of Clinical Neurosciences, University of Oxford, John Radcliffe Hospital, Oxford OX3 9DU, UK

[5] Army Registrar in Neurology and Lecturer in Medicine and Physiology, St Hugh's College, St Margaret's Rd, Oxford OX2 6LE, United Kingdom

[6] Centre for Functional MRI of the Brain, University of Oxford, UK

[7] Current affiliations: Sir Peter Mansfield Imaging Centre, School of Medicine, and National Institute for Health Research (NIHR) Nottingham Biomedical Research Centre, Queens Medical Centre, University of Nottingham, UK

[8] Department of Basic Medical Sciences, College of Veterinary Medicine, Purdue University, West Lafayette, IN, USA

[9] PULSe Interdisciplinary Life Science Program, Purdue University, West Lafayette, IN, USA

[10] Purdue Institute for Integrative Neuroscience, Purdue University, West Lafayette, IN, USA

†Equal contribution authors

*Corresponding authors: antoine.jerusalem@eng.ox.ac.uk; riyi@purdue.edu

Experimental methods

Blast injury exposure

Mild composite blast injury was directed to the head of body-shielded rats as reported previously (1-3). Briefly, a single composite blast was administered to the cranium of body-protected, anesthetised adult male rats using an open-ended shock tube model. The dorsum of the skull was the incident surface first struck by the blast. The exposure conditions under this arrangement had a recorded pressure profile with a near-instantaneous rise to peak pressure, followed by overpressure and underpressure periods as follows: side on (static) 150 kPa maximum overpressure, 1.25 ms overpressure duration, and 20 kPa minimum underpressure; face on (dynamic) 160 kPa maximum overpressure, 1.75 ms overpressure

duration, and 5 kPa minimum underpressure. Rats were positioned outside the shock tube within one tube diameter to maintain shock overpressure conditions (4), as confirmed previously by shadowgraphy high-speed-camera imaging (unpublished data). The rats' heads were restrained to limit gross head motion via a stereotaxic restraint device. These conditions have been validated to result in a mild severity post-injury phenotype (1,5).

Brain deformation recordings

Traces of point deformations, published previously (2) in the living rat brain during blast exposure were used to validate the computational model. Briefly, an implantable soft magnet transducer was placed on the surface of the rat brain prior to blast exposure. A three-member giant magnetoresistance (GMR) reference sensor array was then affixed to the surface of the rat skull. The GMR array was empirically calibrated with a micromanipulator stage to track the soft magnet transducer with 10 µm spatial resolution and 25 kHz temporal resolution. The calibration results were confirmed and replicated with a 2-equation, 3-variable Gaussian computational model (2). The GMR array wirelessly tracked the relative position of the implanted soft magnet in real-time by detecting dynamic changes in the magnetic field. Relative displacements between the surface of the brain and the skull ranging from hundreds of microns up to a millimeter were detected on the surface of the brain using this method under the mild bTBI conditions described above (2).

Acquisition of anatomical and diffusion-weighted magnetic resonance images of the rat brain

T1- and T2-weighted anatomical magnetic resonance imaging (MRI) data were acquired on a 7 Tesla (T) small animal scanner (Bruker Biosystems) using the Paravision 6.0.1 software platform. The same probe (RF RES 300 1H 112/086 QSN TO AD, Bruker Biosystems), gradient coil (BA-GA12SHP BC 70/30), and surface coil (RF SUC 300 1H R BR QSN RO AD, Bruker Biosystems) were used for all image acquisitions. Rat anaesthesia was initiated with 4% isoflurane in air via an inhalation box and anaesthesia delivery system (SomnoSuite Low-Flow Anesthesia System, Kent Scientific). After adequate anaesthetic depth was achieved, the rat was moved to the scanning platform and secured in plastic, MRI-compatible stereotaxic head fixation apparatus (custom made via 3D printing) with bite bar and ear bars. Isoflurane was continuously administered via nosecone between 1-2% to maintain anaesthesia throughout the duration of the scanning procedure. Respiration rate (30-40 breaths/minute) and body temperature (37 ^{0}C) were monitored and maintained via minor anaesthesia and warming surface temperature adjustments throughout the scanning procedure. After positioning the animal in the scanner, wobbling was performed to tune and match the scanner, after which a simple localizer scan was run with B0 adjustment. Upon completion of the localizer, a rapid low-resolution T2 scan was run to visualise the brain in its entirety and incorporate an ellipsoid mapshim procedure. The ellipsoid was aligned with the brain and manually adjusted to encompass only brain tissue while excluding skin, muscle, and skull tissues.

T1- and T2-weighted images were then acquired. For both scan types, a field of view (FOV) of 40 mm × 40 mm × 30 mm (x, y, z where x = lateral, y = dorsoventral, and z = rostrocaudal) was used. The FOV was composed of 512 × 512 × 60 voxels, each of size 0.078 mm × 0.078 mm × 0.5 mm, and was acquired in interleaved coronal slices acquired dorsal to ventral with a FOV saturation pulse used on the ventral aspect of the brain to prevent edge artefacts. T2 scans were acquired with an echo time of 12.615 ms, repetition time of 7308.8 ms, RARE factor of 8, 6 averages, and 1 repetition. T1 scans were acquired with an echo time of 10.625 ms, repetition time of 3215.9 ms, 6 averages, and 1 repetition. While the same animal was still anaesthetised, diffusion-weighted images of the brain were captured using a multi-segment gradient echoplanar imaging (EPI) sequence. The acquisition settings were

adapted from published datasets (6-8). For diffusion-weighted image acquisition, the centre of mass remained aligned with the T1- and T1-weighted images, the FOV was reduced to 36 mm × 18 mm × 27 mm (voxel dimensions 0.2 mm × 0.2mm × 0.5 mm) in order to minimise array size and reduce image acquisition time. Scans were acquired with 5 A0 images (b = 0 s/mm^2; referred heretofore as "B0 volumes") followed by diffusion-weighted images for 30 gradient directions (b = 650 s/mm^2). The entire scan was completed in 6 segments with 2 repetitions. Echo time was 29 ms. Repetition time was 6,500 ms. The gradient duration and dwell time were 3 ms and 10 ms, respectively. Images were visually compared with published datasets and found to be qualitatively similar (6-8). Major fibre tracts with known anatomical orientation (i.e., genu of corpus callosum, cingulate gyrus) were inspected and confirmed to possess anatomically accurate orientations.

Alignment and processing of diffusion-weighted images

All image processing was performed in FSL 5.0.9 (9). Eddy current correction was applied to the raw diffusion image set using the FDT Diffuson Toolbox (10). Brain masking (for both anatomical and diffusion data) was manually performed in FSLView to isolate the brain from surrounding tissues. The masked B0 volumes were extracted from the rest of the diffusion dataset and averaged. This averaged, masked B0 volume was aligned to the masked T1-weighted anatomical reference image using the FLIRT Toolbox (11). Spline-interpolated rigid body (6 parameters) registration was used. The transformation matrix generated by the prior registration was then saved and applied to the entire diffusion dataset (B0 and gradient direction volumes). The diffusion tensor image (DTI) fibre orientation and fractional anisotropy (FA) data were then obtained using the FDT Diffusion Toolbox and "dtifit" function.

Numerical methods

Mechanical behaviour of biological tissues and air

Grey and white matter of brain

The grey matter generally exhibits an isotropic mechanical response (12-13). However, the white matter bundles of axons induce a transversely isotropic mechanical behaviour. In this work, the mechanical behaviour of the grey matter was modelled as isotropic and, the mechanical behaviour of the white matter was modelled by adding the contribution of an anisotropic response associated to the axonal response to the underlying glial matrix (12,14,15). In addition, many authors have observed a strong dependence of both grey and white matter mechanical behaviours on strain rate (13,16). Following these observations, a viscous contribution that incorporates strain rate dependency was added to the constitutive model.

The strain-energy functions from which the stress expressions can be derived read as:

$$\Psi_{\text{iso}} = \Psi_{\text{iso}}^{\text{m}} + \Psi_{\text{iso}}^{\text{a}} \tag{S.1}$$

where $\Psi_{\text{iso}}^{\text{m}}$ and $\Psi_{\text{iso}}^{\text{a}}$ are the strain-energy functions associated to the glial matrix and to the axons respectively, for the white matter, while $\Psi_{\text{iso}}^{\text{a}}$ can simply be discarded for the grey matter. Subsequently, only the white matter model is presented with the assumption that the grey matter model can readily be obtained by considering that $\Psi_{\text{iso}}^{\text{a}} = 0$ and that the grey matter behaves similarly to the white matter glial matrix as a first approximation.

The isotropic glial matrix contribution is defined by using a Gent strain-energy function commonly used in biological tissues (17) and dissociating the strain rate-independent $\Psi_{iso}^{mri}(\mathbf{C}^*)$ from the strain rate-dependent $\Psi_{iso}^{mrd}(\mathbf{C}^{e*})$ contributions:

$$\Psi_{iso}^{m}(\mathbf{C}^*, \mathbf{C}^{e*}) = \Psi_{iso}^{mri}(\mathbf{C}^*) + \Psi_{iso}^{mrd}(\mathbf{C}^{e*}) = -\frac{\mu_m}{2} j_m \ln\left(1 - \frac{I_1^* - 3}{j_m}\right) - \frac{\mu_v}{2} j_v \ln\left(1 - \frac{I_1^{e*} - 3}{j_v}\right) \tag{S.2}$$

where μ_m and μ_v are material parameters associated with the shear modulus, j_m and j_v are dimensionless parameters controlling the limited chain extensibility, $I_1^* = \mathrm{tr}(\mathbf{C}^*)$ is the general deviatoric first strain invariant and $I_1^{e*} = \mathrm{tr}(\mathbf{C}^{e*})$ is the viscous deviatoric first strain invariant. The distortional right Cauchy-Green deformation tensors are defined by $\mathbf{C}^* = (\mathbf{F}^*)^T\mathbf{F}^*$ and $\mathbf{C}^{e*} = (\mathbf{F}^{e*})^T\mathbf{F}^{e*}$, where the distortional part of the deformation gradient is split into elastic and viscous parts as $\mathbf{F}^* = \mathbf{F}^{e*}\mathbf{F}^{v*}$.

To complete the formulation of the glial matrix response, the definition of the viscous flow is needed and its evolution is determined by:

$$\mathbf{L}^v = \dot{\mathbf{F}}^v\mathbf{F}^{-v} = \dot{\gamma}^v\mathbf{N}^v \tag{S.3}$$

where $\mathbf{L}^v$ is the viscous component of the velocity gradient, $\dot{\gamma}^v$ is the viscoelastic multiplier and $\mathbf{N}^v$ is the direction of the viscoelastic flow defined as:

$$\mathbf{N}^v = \frac{\boldsymbol{\sigma}_V^{dev}}{\tau_V} \tag{S.4}$$

where $\boldsymbol{\sigma}_V^{dev}$ is the deviatoric part of $J^{-1}\mathbf{F}^e 2\frac{\partial\Psi_{iso}^{msr}}{\partial\mathbf{C}^e}\mathbf{F}^{eT}$ and $\tau_V = \sqrt{\mathrm{tr}\left(\boldsymbol{\sigma}_V^{dev}\boldsymbol{\sigma}_V^{dev}\right)}$ is the effective stress driving the viscous flow. The rate equation for viscous flow is given by Prevost et al. (18):

$$\dot{\gamma}^v = \dot{\gamma}_0^v f_R \left(\frac{\sqrt{\boldsymbol{\sigma}_V^{dev}:\boldsymbol{\sigma}_V^{dev}}}{\sqrt{2}\sigma_{VT}}\right)^n \tag{S.5}$$

where

$$f_R = \frac{1}{\left(\alpha + \sqrt{\mathrm{tr}(\mathbf{F}^{v*}(\mathbf{F}^{v*})^T)/3} - 1\right)^C} \tag{S.6}$$

and where $\dot{\gamma}_0^v$ is a dimensional scaling constant, $0 < \alpha \ll 1$ is a constant incorporated to eliminate singularity and σ_{VT}, C and n are material properties.

The free energy associated to the axonal contribution is defined by the free energy function proposed by Nolan et al. (19):

$$\Psi_{iso}^{a}\left(\mathbf{C}^*, \widehat{\mathbf{A}}_0\right) = \frac{k_1}{2k_2}\{\exp[k_2(I_4^* - 1)^2] - 1\} \tag{S.7}$$

where k_1 and k_2 are material parameters quantifying the increase of stiffness in the axonal direction. The deviatoric fourth strain invariant $I_4^* = \mathrm{tr}\left(\widehat{\mathbf{A}}_0\mathbf{C}^*\right)$ is defined depending on a structure tensor $\widehat{\mathbf{A}}_0$, in turn function of a single dispersion parameter ξ and the preferred axon orientation $\hat{\mathbf{a}}_0$. Gasser et al. proposed the following compact form (20):

$$\widehat{\mathbf{A}}_0 = \xi\mathbf{I} + (1 - 3\xi)\hat{\mathbf{a}}_0 \otimes \hat{\mathbf{a}}_0 \tag{S.8}$$

The definition of the structure tensor presented in Equation (S.8) has been previously used by Wright et al. to incorporate neural tract alignment through DTI (21). Following the same approach, a functional dependence on FA is proposed for the dispersion parameter ξ:

$$\xi = \frac{1}{2}\frac{-6+4FA^2+2\sqrt{3FA^2-2FA^4}}{-9+6FA^2} \tag{S.9}$$

When ξ adopts a value of 1/3, an isotropic distribution of the axons is considered and the structure tensor is spherical. When ξ adopts a value of 0, an ideal coalignment of the axons is considered and the structure tensor reduces then to $\widehat{\mathbf{A}}_{\mathbf{0}} = \hat{\mathbf{a}}_{\mathbf{0}} \otimes \hat{\mathbf{a}}_{\mathbf{0}}$. Thus, the anisotropic behaviour of the white matter arising from axonal orientation and axonal dispersion can be taken into account in the constitutive modelling through $\widehat{\mathbf{A}}_{\mathbf{0}}$ by connecting experiments with modelling.

Using the methodology followed in Garcia-Gonzalez et al. (22), and noting that $\boldsymbol{\sigma}_{\mathbf{iso}} = 2J^{-1}\mathbf{F}\frac{\partial \Psi_{iso}^{mri}}{\partial \mathbf{C}}\mathbf{F}^{\mathbf{T}} + 2J^{-1}\mathbf{F}^{e}\frac{\partial \Psi_{iso}^{mrd}}{\partial \mathbf{C}^{e}}\mathbf{F}^{\mathbf{eT}} + 2J^{-1}\mathbf{F}\frac{\partial \Psi_{iso}^{a}}{\partial \mathbf{C}}\mathbf{F}^{\mathbf{T}}$, the following expression for the isochoric Cauchy stress tensor is obtained:

$$\boldsymbol{\sigma}_{\mathbf{iso}} = \frac{\mu_m}{J}\frac{1}{1-\frac{I_1^*-3}{j_m}}\operatorname{dev}(\mathbf{B}^*) + \frac{\mu_v}{J}\frac{1}{1-\frac{I_1^{e*}-3}{j_v}}\operatorname{dev}(\mathbf{B}^{e*}) + \frac{2k_1}{J}(I_4^*-1)\exp[k_2(I_4^*-1)^2]\operatorname{dev}(\mathbf{F}^*\widehat{\mathbf{A}}_{\mathbf{0}}\mathbf{F}^{*\mathbf{T}}) \tag{S.10}$$

where $\operatorname{dev}(\mathbf{B}^*)$ and $\operatorname{dev}(\mathbf{B}^{e*})$ are the deviatoric parts of the distortional left Cauchy-Green deformation tensors $\mathbf{B}^* = \mathbf{F}^*(\mathbf{F}^*)^T$ and $\mathbf{B}^{e*} = \mathbf{F}^{e*}(\mathbf{F}^{e*})^T$.

Finally, the volumetric response of the tissue is defined by using the Tait equation of state (EOS), commonly used to model fluids and brain tissue (23):

$$P = B\left[\left(\frac{\rho}{\rho_o}\right)^{\Lambda_o+1} - 1\right] \tag{S.11}$$

where B and Λ_o are material constants and ρ_o is the reference density. The parameter B can be computed through its relation with the reference bulk modulus K_o:

$$K_o = B(\Lambda_o + 1) \tag{S.12}$$

This constitutive model was calibrated against experimental data for grey and white matter with a good agreement between experiments from literature and model predictions for both quasi-static and very high rate loadings, see Fig. S5 (13). Note that the strain rate sensitivity is accounted for by the isotropic response of the glial matrix. Also, as observed elsewhere (13,16), anisotropy due to axonal orientation is not relevant at high rates since the stiffening effect due to strain rate effects plays the predominant role in the mechanical behaviour of the tissue. The anisotropic parameters were defined following the work of Wright et al. (21) by assuming the same ratio of the fibre reinforcement parameter to the quasi-static shear modulus (k_1/μ_m) as in the fit proposed by Velardi et al. (12). Finally, the volumetric parameters were taken from the work of Moore et al. (23). The model parameters for both grey and white matters are provided in Table S1. Note that, due to the lack of available data for human or rat tissue at the high strain rates reached during blast events, the model was calibrated with porcine tissue. Therefore, although the tendencies and overall response of the tissue can be assumed the same, some quantitative differences are expected.

Skin/fat and scalp

The mechanical behavior of skin/fat and scalp was defined herein following Equations (1) and (2) (main text) for the volumetric contribution and using a Neo-Hookean strain-energy function the isochoric stress response:

$$\Psi_{iso}(\mathbf{C}^*) = \frac{\mu}{2}(I_1^* - 3) \quad \text{(S.13)}$$

where μ is the shear modulus. The expression for the isochoric Cauchy stress tensor can be obtained by noting that $\boldsymbol{\sigma}_{\mathbf{iso}} = 2J^{-1}\mathbf{F}\frac{\partial\Psi_{iso}(\mathbf{C}^*)}{\partial\mathbf{C}}\mathbf{F}^{\mathbf{T}}$:

$$\boldsymbol{\sigma}_{\mathbf{iso}} = \frac{\mu}{J}\operatorname{dev}(\mathbf{B}^*) \quad \text{(S.14)}$$

The material parameters for skin/fat and scalp tissues are provided in Table S2. These constants were taken from the literature (23-25).

Skull

The mechanical response of the skull was described by several authors as isotropic and linear elastic as a first approximation (24,26,27). However, its shock response has also been previously described with nonlinear model, e.g., Moore et al. (23) defined the volumetric response of the skull by using a Mie-Grüneisen EOS. Here, as the deformations observed in the simulations within the skull are very small, the volumetric stress response of this tissue was simply defined by a linear approximation as:

$$\boldsymbol{\sigma}_{\mathbf{vol}} = K_o(J - 1)\mathbf{I} \quad \text{(S.15)}$$

The material parameters for skull were taken from previous work and are provided in Table S3 (24).

CSF and ventricles

The isochoric mechanical behavior of CSF and ventricles was considered as driven by its water content and thus characterized by a Newtonian viscosity:

$$\boldsymbol{\sigma}_{\mathbf{iso}} = 2\eta\dot{\boldsymbol{\varepsilon}}' \quad \text{(S.16)}$$

where η is the dynamic viscosity and $\dot{\boldsymbol{\varepsilon}}'$ is the deviatoric strain rate.

The volumetric response is defined here through the Mie-Grüneisen EOS:

$$P = \frac{\rho_o c_o^2 \varsigma}{(1 - s\varsigma)^2}\left(1 - \frac{\Gamma_o \varsigma}{2}\right) + \Gamma_o \rho_o E_m \quad \text{(S.17)}$$

where $\varsigma = 1 - \rho_o/\rho$ is the nominal volumetric compressive strain with ρ_o as the initial density. c_o is the speed of sound in water, s is the slope of the u_s-u_p curve in the Hugoniot formulation, where u_s and u_p are the shock and particle velocities, respectively. Γ_o is the Grüneisen coefficient and E_m is the internal energy per unit mass.

The material parameters for CSF and ventricles were identified from literature and are provided in Table S4 (24).

Air

The isochoric mechanical behavior of air was defined with a Newtonian viscosity following Equation (S.16), while the volumetric response was defined by the ideal gas EOS:

$$P + P_a = \rho_o RT \tag{S.18}$$

where P_a is the atmospheric pressure, R the gas constant and T the absolute temperature. To complete the definition of the volumetric response, the specific energy E is defined as:

$$E = E_o + \int_{T_o}^{T} C_v \, dT \tag{S.19}$$

where E_o and T_o are the specific energy and temperature in the initial state, and C_v is the specific heat at constant volume.

The material parameters for air were taken from the literature and are provided in Table S5 (28).

Injury criteria

The maximum of the following quantities for the whole duration of the simulations was calculated. The simulation time was taken to be long enough so as to ensure that the maximum was reached.

Pressure

The pressure criterion is based on the maximum value of P, Equation (1) of the main text, reached in each region during the deformation process.

Von Mises stress

The von Mises stress can be derived from Equation (1) of the main text as:

$$\boldsymbol{\sigma}_{\mathbf{VM}} = \sqrt{\sigma_{11}^2 + \sigma_{22}^2 + \sigma_{33}^2 - (\sigma_{11}\sigma_{22} + \sigma_{22}\sigma_{33} + \sigma_{33}\sigma_{11}) + 3(\sigma_{12}^2 + \sigma_{23}^2 + \sigma_{31}^2)} \tag{S.20}$$

Equivalent strain

The equivalent strain is defined as:

$$\bar{\varepsilon} = \sqrt{\frac{2}{3}\mathbf{E}:\mathbf{E}} \tag{S.21}$$

where $\mathbf{E} = \frac{1}{2}(\mathbf{C} - \mathbf{I})$ is the Green-Lagrangian strain tensor.

Shear energy rate

The shear energy rate per unit volume $\dot{\Psi}_{iso}$ is the time derivative of Ψ_{iso}. In this work, the deviatoric energy was computed following Equation (S.1).

Volumetric energy rate

The volumetric energy rate per unit volume $\dot{\Psi}_{vol}$ is the time derivative of Ψ_{vol}, defined by Equation (S.11):

$$\Psi_{vol} = \int P \frac{d\rho}{\rho} \tag{S.22}$$

Axonal stretch

The axonal stretch can be defined from the fourth strain invariant as:

$$\bar{\lambda}_{axon} = \sqrt{\mathrm{I}_4^*} \tag{S.23}$$

Axonal stretch energy rate

The energy rate from axonal deformation $\dot{\Psi}_{\mathrm{axon}}$ is the time derivative of Ψ_{axon}, defined by taking into account the evolution of the product of the stress tensor with the rate of deformation tensor, projected along the axonal direction:

$$\Psi_{\mathrm{axon}} = \int (\tilde{\boldsymbol{\sigma}}_{\mathbf{iso}} \colon \widehat{\mathbf{A}}_{\mathbf{o}})(\tilde{\mathbf{d}} \colon \widehat{\mathbf{A}}_{\mathbf{o}})\, \mathrm{dt} \tag{S.24}$$

where $\tilde{\boldsymbol{\sigma}}_{\mathbf{iso}} = \mathbf{R}^{\mathbf{T}}\boldsymbol{\sigma}_{\mathbf{iso}}\mathbf{R}$ and $\tilde{\mathbf{d}} = \mathbf{R}^{\mathbf{T}}\mathbf{dR}$ are, respectively, the corotational Cauchy stress and the corotational rate of deformation tensor, and where $\mathbf{d}$ is the symmetrical part of the total velocity gradient $\mathbf{l} = \dot{\mathbf{F}}\mathbf{F}^{-1}$. $\mathbf{R} = \mathbf{F}\mathbf{U}^{-1}$ is the rotation tensor, where $\mathbf{U}$ is the stretch tensor.

Simplified FEHM

The simplified FEHM was designed as a combination of concentric spheres representing: skull, CSF, brain and ventricles. In addition, another layer was incorporated to introduce a protective shield on the head (see Fig. S7).

Role of acoustic impedance and impulse mitigation for blast protective devices

When a blast wave reaches the head-shield interface, the incident stress σ_{i} is conserved into a reflected stress σ_{r} and a transmitted stress σ_{t}:

$$\sigma_{\mathrm{t}} = \sigma_{\mathrm{i}} + \sigma_{\mathrm{r}} \tag{S.25}$$

Similarly, by compatibility at the interface, the transmitted particles velocity $\mathrm{v_t} = \frac{\sigma_{\mathrm{t}}}{\sqrt{\mathrm{E_h}\rho_{\mathrm{h}}}}$ can be obtained from the incident particles velocity and the reflected particles velocity $\mathrm{v_r}$:

$$\mathrm{v_t} = \mathrm{v_i} - \mathrm{v_r} \tag{S.26}$$

where $\mathrm{E_h}$ and ρ_{h} are respectively the Young's modulus and the density of the head (assuming the head homogenised).

By combining Equations (S.25) and (S.26), the transmitted stress can be expressed as:

$$\sigma_t = \frac{2\sqrt{E_h\rho_h}}{\sqrt{E_s\rho_s}+\sqrt{E_h\rho_h}}\sigma_i \tag{S.27}$$

where E_s is the Young's modulus of the shield and ρ_s is the density of the shield. The reduction in the stress amplitude can be thus controlled through the acoustic impedance of the shield material $\sqrt{E_s\rho_s}$.

Assuming a perfectly-elastic collision as a first approximation, the transmitted impulse I_h can be obtained from the combination of the conservation of momentum and the conservation of energy equations as:

$$I_h = I_o \frac{2\frac{m_h}{m_s}}{1+\frac{m_s}{m_h}} \tag{S.28}$$

where m_h and m_s are the masses of the head and the shield, respectively, V_h is the head velocity after the blast and I_o is the impulse imparted to the shield (29). Therefore, impulse mitigation can be reached by increasing the shield mass. Alternatively, energy dissipation can be reached by additional consideration of inelastic deformation in the shield.

References

1. M. Walls, N. Race, L. Zheng, S.M. Vega-Alvarez, G. Acosta, J. Park, R. Shi, Structural and biochemical abnormalities in the absence of acute deficits in mild primary blast-induced head trauma. J Neurosurg 124(3), 675-686 (2015).

2. S. Song, N.S. Race, A. Kim, T. Zhang, R. Shi, B. Ziaie, A Wireless Intracranial Brain Deformation Sensing System for Blast-Induced Traumatic Brain Injury. Sci Rep 5, 16959 (2015).

3. N.S. Race, J. Lai, R. Shi, E.L. Bartlett, Differences in post-injury auditory system pathophysiology after mild blast and non-blast acute acoustic trauma. J Neurophysiol, jn-00710 (2017).

4. A.J. Newman, J.C. Mollendorf, The peak overpressure field resulting from shocks emerging from circular shock tubes. J Fluids Eng 132(8), 081204 (2010).

5. N.S. Race, E.L. Lungwitz, S.V. Alvarez, G. Acosta, T.R. Warner, K. Andrews, J Cao, K.H. Lu, Z. Liu, A. Shekhar, W.A. Truitt, R. Shi, Psychosocial dysfunction after mild blast TBI: toward a neurobiological basis. Near submission, draft available upon request (2017).

6. M.G. Preti, A. Di Marzio, A. Mastropietro, D. Aquino, G. Baselli, M.M. Laganà, I. Zucca, C. Frassoni, R. Spreafico, Tractographic reconstruction protocol optimization in the rat brain invivo: towards a normal atlas. In Engineering in Medicine and Biology Society, EMBC, Annual International Conference of the IEEE 2011, 8467-8470 (2011).

7. M. Figini, I. Zucca, D. Aquino, P. Pennacchio, S. Nava, A. Di Marzio, M.G. Preti, G. Baselli, R. Spreafico, C. Frassoni, In vivo DTI tractography of the rat brain: an atlas of the main tracts in Paxinos space with histological comparison. Magn Reson Imaging 33(3), 296-303 (2015).

8. L.A. Harsan, C. Dávid, M. Reisert, S. Schnell, J. Hennig, D. von Elverfeldt, J.F. Staiger, Mapping remodeling of thalamocortical projections in the living reeler mouse brain by diffusion tractography. Proc Natl Acad Sci 110(19), E1797-806 (2013).

9. S.M. Smith, Fast robust automated brain extraction. Human Brain Mapp 17 (3), 143–155 (2002).

10. J.L.R. Andersson, S.N. Sotiropoulos, An integrated approach to correction for off-resonance effects and subject movement in diffusion MR imaging. NeuroImage 125, 1063–1078 (2016).

11. M. Jenkinson, S.M. Smith, A global optimisation method for robust affine registration of brain images. Med Image Anal 5(2), 143-156 (2001).

12. F. Velardi, F. Fraternali, M. Angelillo, Anisotropic constitutive equations and experimental tensile behavior of brain tissue. Biomech Model Mechan 5, 53–61 (2006).

13. F. Pervin, W.W. Chen, Dynamic mechanical response of bovine gray matter and white matter brain tissues under compression. J Biomech 42, 731–735 (2009).

14. Y. Feng, R.J. Okamoto, R. Namani, G.M. Genin, P. Bayly, Measurements of mechanical anisotropy in brain tissue and implications for transversely isotropic material models of white matter. J Mech Behav Biomed Mater 23, 117–132 (2013).

15. X. Ning, Q. Zhu, Y. Lanir, S.S. Margulies, A transversely isotropic viscoelastic constitutive equation for brainstem undergoing finite deformation. J Biomech Eng. 128, 925–933 (2006).

16. X. Nie, B. Sanborn, T. Weerasooriya, W. Chen, High-rate bulk and shear responses of bovine brain tissue. Int J Impact Eng 53, 56-61 (2013).

17. A.N. Gent, A New Constitutive Relation for Rubber. Rubber Chem Technol 69(1), 59–61 (1996).

18. T.P. Prevost, G. Jin, M.A. de Moya, H.B. Alam, S. Suresh, S. Socrate, Dynamic mechanical response of brain tissue in indentation in vivo, in situ and in vitro. Acta Biomater 7(12), 40904101 (2011).

19. D.R. Nolan, A.L. Gower, M. Destrade, R.W. Ogden, J.P. McGarry, A robust anisotropic hyperelastic formulation for the modelling of soft tissue. J Mech Behav Biomed 39, 48–60 (2014).

20. T.C. Gasser, R.W. Ogden, G.A. Holzapfel, Hyperelastic modelling of arterial layers with distributed collagen fibre orientations. J Roy Soc Interface 3, 15–35 (2006).

21. R.M. Wright, A. Post, B. Hoshizaki, K.T. Ramesh, A multiscale computational approach to estimating axonal damage under inertial loading of the head. J Neurotraum 30, 102–118 (2013).

22. D. Garcia-Gonzalez, A. Jerusalem, S. Garzon-Hernandez, R. Zaera, A. Arias, A continuum mechanics constitutive framework for transverse isotropic soft tissues. J Mech Phys Solids112, 209–224 (2018).

23. D.F. Moore, A. Jérusalem, M. Nyein, L. Noels, M.S. Jaffee, R.A. Radovitzky, Computational biology – modeling of primary blast effects on the central nervous system. Neuroimage 47(S2), 10–20 (2009).

24. D. Garcia-Gonzalez, J. Jayamohan, S.N. Sotiropoulos, S.-H. Yoon, J. Cook, C.R. Siviour, A. Arias, A. Jérusalem, On the mechanical behaviour of PEEK and HA cranial implants under impact loading. J Mech Behav Biomed Mater 69, 342-354 (2017).

25. T.J. Horgan, M. Gilchrist, The creation of three-dimensional finite element models for simulating head impact biomechanics. Int J Crashworthiness 8, 353–366 (2003).

26. F. El Halabi, J.F. Rodriguez, L. Rebolledo, E. Hurtós, M. Doblaré, Mechanical characterization and numerical simulation of polyether-ther-ketone (PEEK) cranial implants. J Mech Behav Biomed Mater 4, 1819–1832 (2011).

27. D. Sahoo, C. Deck, R. Willinger, Development and validation of an advanced anisotropic visco-hyperelastic human brain FE model. J Mech Behav Biomed Mater 33, 24–42 (2014).

28. G.A. Christou, L.R. Young, R. Goel, A.P. Vechart, A. Jérusalem, Shock attenuation of PMMA sandwich panels filled with soda-lime glass beads: A fluid-structure interaction continuum model simulation. Int J Impact Eng 47, 48-59 (2012).

29. T. Rahimzadeh, E.M. Arruda, M.D. Thouless, Design of armor for protection against blast and impact. J Mech Phys Solids 85, 98–111 (2015).

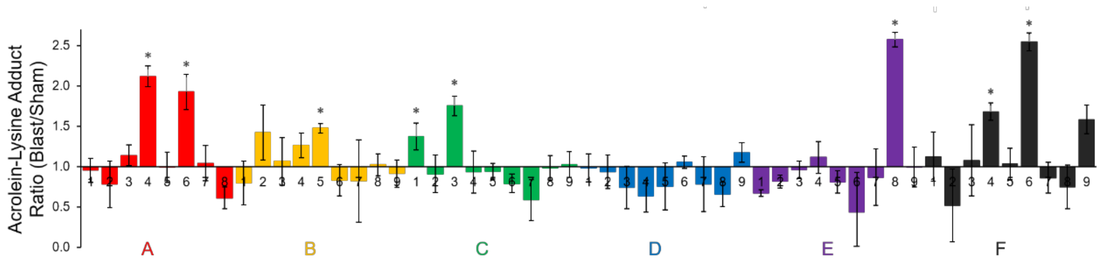

Fig. S1: Forebrains of Sham and Blast animals at 24 hours post-injury (coinciding with peak urine 3-HPMA elevation) were subdivided into 53 sub-regions for acrolein-lysine adduct immunoblotting analysis. Overall, 8 out of 53 regions demonstrated significant increases in acrolein-lysine adduct immunoreactivity after mild b-TBI when compared to sham-injured animals. Areas with significantly higher oxidative stress relative to Sham included those containing the OFC and agranular insula bilaterally (A4 + A6), the anterior striatum (B5), bilateral somatosensory cortices (C1 + C3), the caudal hypothalamus + rostral midbrain (E8), and areas containing the auditory cortex + temporal association cortex + hippocampal CA2 bilaterally (F4+F6). The anatomical positions of these regions are illustrated in Fig. 1 of the main text. * $p<0.05$. Unpaired t-tests. Data presented as mean ± SEM. N=4/group.

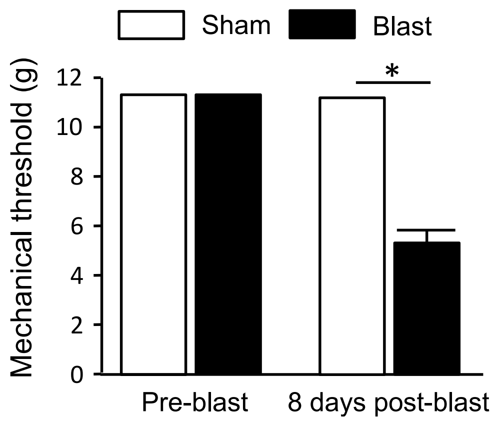

Fig. S2: Behavioural facial hypersensitivity following mild bTBI. Mechanical periorbital allodynia was assessed before mild bTBI induction and 8 days post-mild bTBI. Before mild bTBI induction, both sham and blast groups show no difference in facial mechanical threshold; however, following mild bTBI the blast group shows a significant decrease in mechanical threshold compared to sham animals, indicating facial hypersensitivity compared to the sham group. * $p<0.05$. One-way ANOVA and Tukey test. Data presented as mean ± SEM. N=8/group.

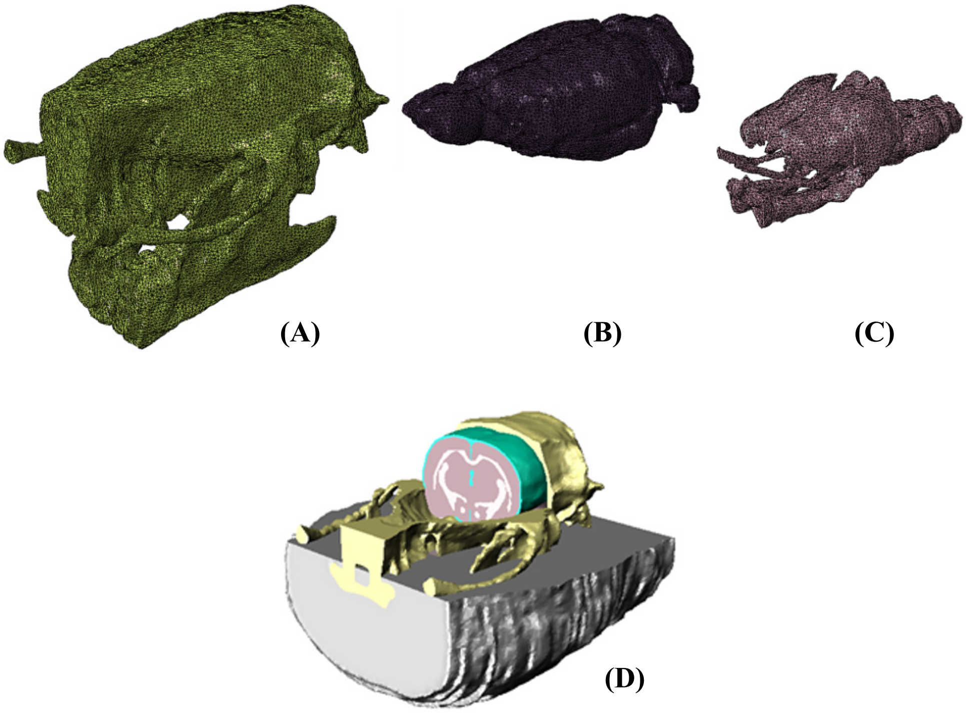

Fig. S3. Full rat head finite element model showing various components: (A) skull; (B) grey matter; (C) white matter. The whole model also includes skin/fat and cerebrospinal fluid (D).

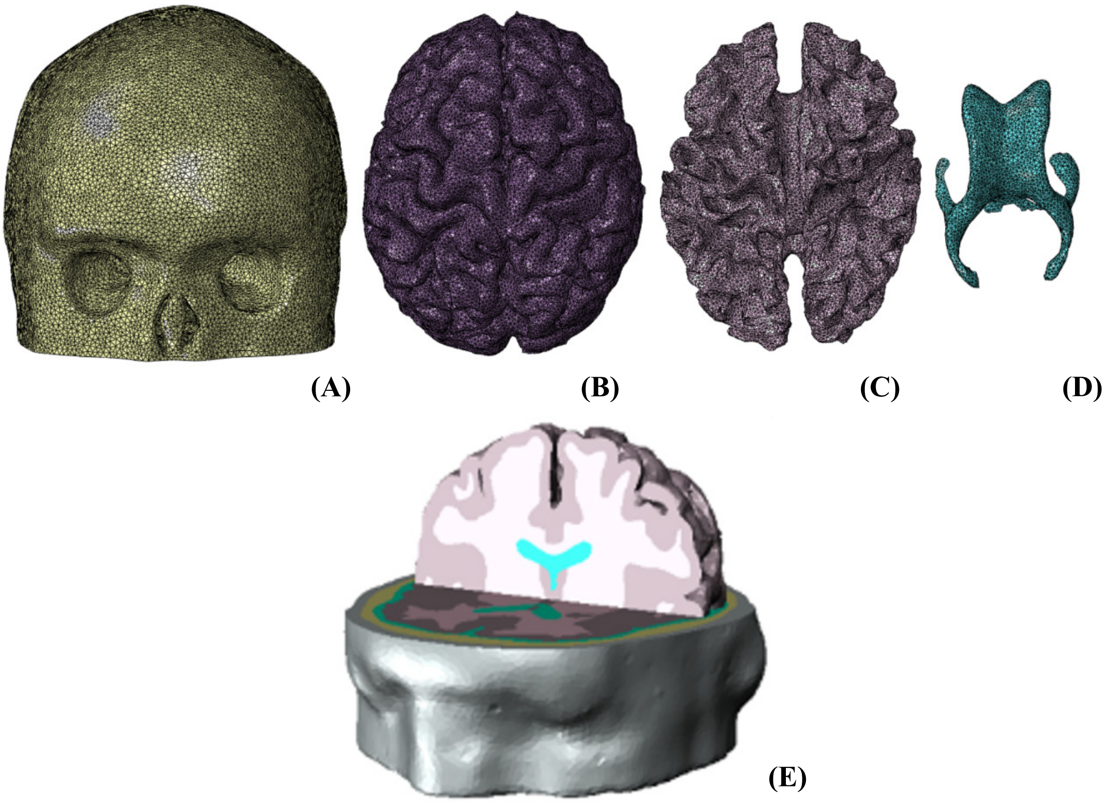

FIG. S4. Full human head finite element model showing various components: (A) skull; (B) grey matter; (C) white matter; (D) ventricles. The whole model also includes skin/fat and cerebrospinal fluid (E).

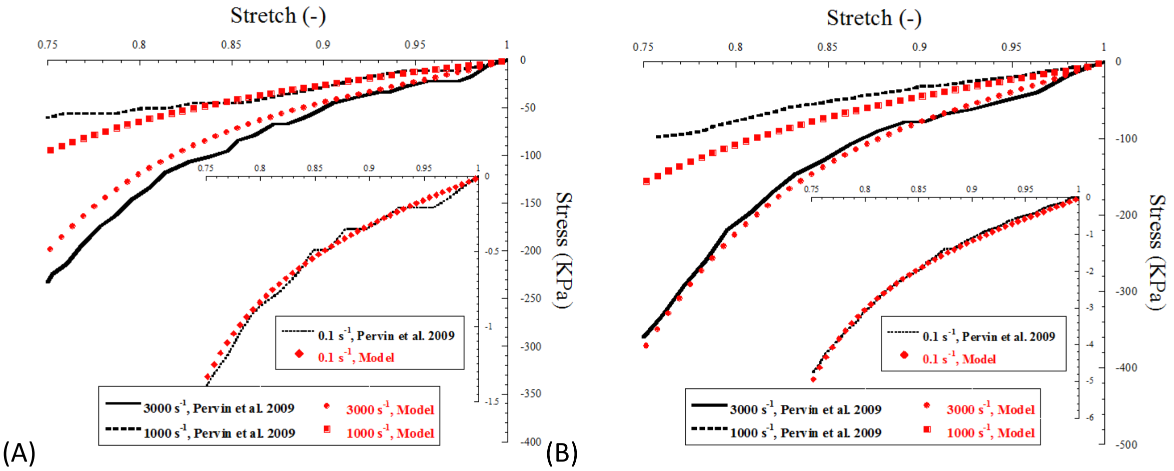

Fig. S5. Stress-stretch experimental curves of brain tissue at different strain rates (23) versus model predictions: (a) grey matter; (b) white matter.

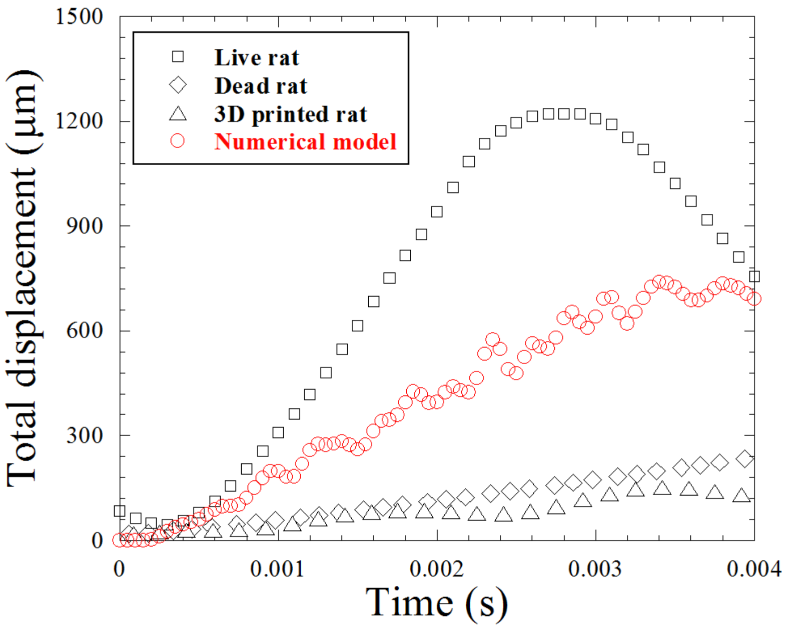

Fig. S6: Comparison between brain displacement during the blast exposure predicted by the model and experimental data (2).

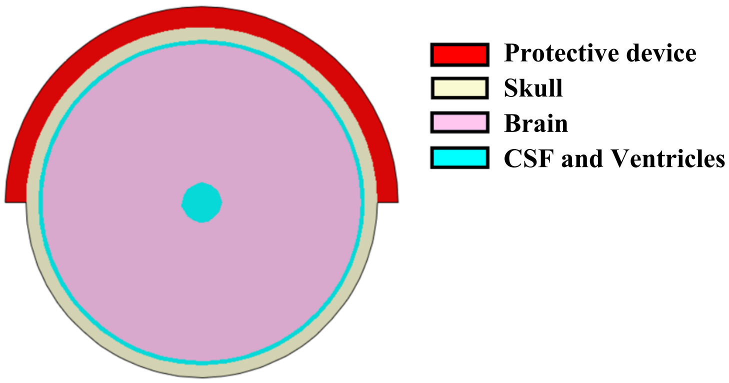

Fig. S7: Simplified FEHM incorporating a protective shield.

Table S1: Material parameters for grey and white matters of brain.

Tissue	Volumetric response			Axonal shear response	
	ρ_o (kg/m^3)	K_o (GPa)	Λ_o	k_1 (kPa)	k_2 (Pa)
Grey matter	1040	2.19	6.15	-	-
White matter	1040	2.19	6.15	2.14	0.0

Tissue	Grey matter/white matter glial matrix shear response								
	μ_m (kPa)	j_m	μ_v (kPa)	j_v	$\dot{\gamma}_o^v$ (s^{-1})	n	σ_{VT} (kPa)	C	α
Grey matter	1.2	0.5	280	0.13	0.1	1.3	0.6	1.6	0.03
White matter	5	0.5	450	0.1	0.1	1.3	0.6	1.3	0.03

Table S2: Material parameters for skin/fat and scalp.

Tissue	Volumetric response			Shear response
	ρ_o (kg/m^3)	K_o (GPa)	Λ_o	μ (MPa)
Skin/fat and Scalp	1100	34.7	6.15	5.88

Table S3: Material parameters for skull.

Tissue	Volumetric response		Shear response
	ρ_o (kg/m^3)	K_o (GPa)	μ (GPa)
Skull	1728	4.76	3.28

Table S4: Material parameters for CSF and ventricles.

Tissue	Volumetric response				Shear response
	ρ_o (kg/m^3)	c_o (m/s)	s	Γ_o	η (Pa s)
CSF and ventricles	1000	1450	1.99	0.11	0.00089

Table S5: Material parameters for air.

Tissue	Volumetric response				Shear response
	ρ_o (kg/m^3)	R (J/kg K)	C_v (J/kg K)	P_a (kPa)	η (Pa s)
Air	1.208	287.04	718.16	101.325	$1.82\cdot10^{-5}$
